# Deep neck infection after third molar extraction: A case report

**DOI:** 10.15171/joddd.2017.030

**Published:** 2017-09-20

**Authors:** Alberto Ferreira da Silva Junior, Gustavo Silvestre de Magalhaes Rocha, Camila Fialho da Silva Neves de Araujo, Ademir Franco, Rhonan Ferreira Silva

**Affiliations:** ^1^Maxillofacial Surgery and Traumatology of the Emergency Hospital of Goiania (HUGO), Goias, Brazil; ^2^Maxillofacial Surgery, Paulista University, Goias, Brazil; ^3^Scientific Consultant, Curitiba, Brazil; ^4^Forensic Odontology, Paulista University, Goias, Brazil; ^5^Forensic Odontology, Federal University of Goias, Brazil

**Keywords:** Abscess, infection, extraction, neck, third molar

## Abstract

Deep neck infections are associated with high morbidity rates in dentistry. Early diagnosis and intervention play an essential part in decreasing morbidity rates. The present study aims to report a case of odontogenic deep neck infection after third molar extraction. A 51-year-old male patient underwent extraction of the mandibular right third molar. Seven days later, the patient developed symptoms and signs of progressive infection. Laboratorial and radiologic examinations in association with clinical investigations confirmed deep neck infection. Extraoral drainage was performed under orotracheal intubation. Postoperative laboratory tests and clinical examinations revealed signs of complete remission within a follow-up period of 10 days. Considering the invasive nature of pathogens related to deep neck infections, it is possible to infer that a combination of accurate diagnosis and early intervention plays an essential role in the field of maxillofacial surgery and pathology.

## Introduction

Odontogenic infections may emerge as postoperative complications after dissemination of dental and periodontal pathogens. Mostly, these infections are restricted to the dentomaxillofacial area. However, the involvement of deep cervical spaces eventually occurs.

Surveys on the progression of odontogenic infections to deep spaces of the neck were performed during the past decade, indicating high prevalence rates. Eftekharian et al^1^ evaluated 112 files of patients with deep neck infections and reported a prevalence rate of 31.3%. Boscolo-Rizzo et al^2^ analyzed 297 patients with deep neck infections of known origin, detecting a prevalence rate of 27.9%. Furthermore, cervical infections may rapidly progress, descending into the thorax and abdomen. Consequently, life-threatening conditions, involving mediastinitis^3^ and even pelvic infections,^4^ are established, raising the mortality rates up to 50%.^5,6^ As a result, immediate medical interventions are necessary.

Considering the close association between dental treatments and high morbidity, the management of deep neck infections arouses the interest of general dental professionals, maxillofacial surgeons, stomatologists and radiologists. In this context, the present study reports a case of deep neck infection after tooth extraction, highlighting the clinical importance of early diagnosis and intervention in dentistry.

## Case report

A 51-years-old male patient underwent extraction of mandibular right third molar (#48). Seven days after the surgery, the patient developed facial edema, fever, intraoral purulent discharge and extreme local pain. The patient was referred to the Department of Maxillofacial Surgery and Traumatology of the Emergency Hospital of Goiania, State of Goias, Brazil.

**Figure 1:**
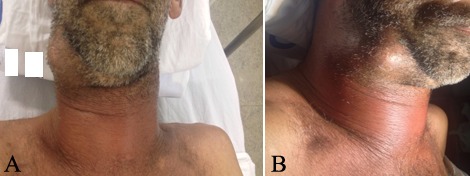
Preoperative clinical presentation of the edema in the right submandibular region in frontal (A) and lateral views (B).

**Figure 2:**
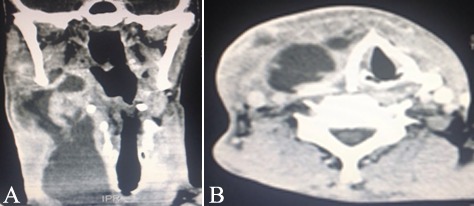
Preoperative computed tomography in coronal (A) and axial (B) views, revealing infectious cavities in the deep neck.

During admission, the anamnesis did not reveal systemic pathologies, chronic use of medical drugs or potential medical allergies. Yet clinically, an evident edema was detected in the right submandibular region presenting a central floating area (Figure 1). Infectious cavities in the right and left submandibular, pterygomandibular and pharyngeal regions were observed through computed tomography scans (Figure 2). Laboratory blood tests revealed levels of hematocrit (36.2%), hemoglobin (11.9 g/dL); leukocytes (24500/mm^3^), gram-negative rods (8%), C-reactive protein (18.15 mg/L) and erythrocyte sedimentation rate (20 mm/h) (Table 1). Based on the clinical, radiographic and laboratory examinations, the patient was diagnosed with an odontogenic deep neck abscess.

**Table 1:** Results of blood tests performed to follow the patient’s condition during the five days of hospitalization

**Blood parameters**	**Postoperative days of hospitalization**
	1st	2nd	3rd	5th
**Hematocrit (%)**	36.2	32.6	34.2	34.8
**Hemoglobin (g/dL)**	11.9	10.6	11.1	11.3
**Leukocytes (mm³)**	24.500	7.000	7.400	9.200
**Gram-negative rods (%)**	8	1	4	2

**Figure 3:**
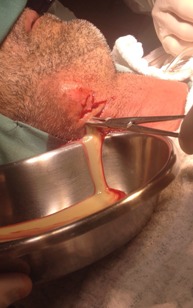
Intraoperative view of the purulent content of the lesion.

**Figure 4:**
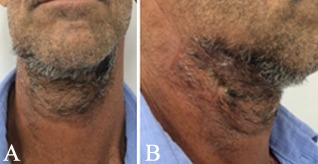
Postoperative follow-up of 10 days.

The therapeutic intervention consisted of surgical drainage through an extraoral incision in the submandibular floating area (Figure 3). The procedure was performed under general anesthesia and endotracheal intubation. The incision was followed by digital dissection in the anteroposterior and craniocaudal directions. Two #2 Penrose’s drains and one #1 Penrose’s drain were installed on the right submandibular region, while a single #1 Penrose’s drain was installed in the left submandibular region. A total volume of approximately 200 mL of purulent content was drained from the submandibular region. The patient was medicated with intravenous clindamycin (600 mg, every 6 hours, for 6 days). During this period new blood tests were performed (Table 1). Moreover, the patient presented better conditions, allowing the removal of drains. Follow-up was performed 10 days after the surgical intervention, revealing no sequelae or signs of infection (Figure 4).

## Discussion

Third molar extraction is not considered a procedure of major complexity in the field of maxillofacial surgery.^7^ However, postoperative complications, such as alveolitis, paresthesia and infection, might prove challenging situations for clinical management.^8^ In particular, odontogenic infections related to the extraction of third molars are classified according to morphological location, such as peritonsillar, pharyngeal and submandibular infections.^9^ The severity of these infections increases with a lack of adequate treatment, potentially evolving into life-threatening morbidities, such as mediastinitis.^9^

Santos Gorjón et al^9^ performed a large descriptive review of cases of deep neck infections, reporting that most of the patients exhibited involvement of peritonsillar and submandibular areas. In detail, the authors also revealed that submandibular abscesses were associated with odontogenic infections in 60% of the adult patients. Similarly in the presented case, tomographic exams revealed the involvement of multiple deep neck infectious cavities, mainly in the submandibular and pharyngeal spaces. Another large-population survey was performed by Boscolo-Rizzo et al,^2^ revealing that neck swelling and throat pain were reported by 93.2% and 56.2% of the patients with deep neck infections, respectively. Similarly, these clinical findings were also the main evidence observed in the present case. Additionally, the authors reported that 15 patients had Ludwig’s Angina, characterizing diffuse gangrenous cellulitis of the submandibular and sublingual spaces.^2^ On the other hand, in our case the patient did not exhibit involvement of the sublingual space.

Despite the absence of Ludwig’s Angina, surgical intervention was essentially necessary to stop the progression of the infection, consequently preventing mediastinitis and pericarditis.^10^ Staffieri et al^10^ investigated 282 records of patients with deep neck infections, revealing that surgical intervention was performed in 184 patients (65.2%). Most of the surgeries consisted of intraoral incision and drainage, surgical exploration and drainage, and dental extraction.^10^ The remaining patients were treated with intravenous antibiotic medication alone. Based on the literature, a combination of intravenous antibiotics and extraoral neck drainage was used as a therapeutic medical intervention. In this context, the medical literature also indicates the microbiological culture of pathogens to support more accurate drug prescriptions. In the study by Staffieri et al^10^ *Streptococcus viridans* group was found to be the most prevalent pathogen, followed by gram-positive anaerobic cocci and *Staphylococcus epidermidis*. Bottin et al^5^ (2003) revealed a high prevalence of *Peptostreptococcus* sp., and *Streptococcus viridans*, indicating a potential relation with odontogenic infection. Yet Boyanova et al^11^ investigated the anaerobic flora of patients with deep infections of the head and neck region, showing that the most prevalent pathogens were *Prevotella*, *Fusobacterium* species, *Actinomyces* spp., anaerobic cocci, and *Eubacterium* spp. The present study does not comprise the results of microbiological culture, which could aid the treatment and prognosis. However, a combination of extraoral drainage and clindamycin proved a proper approach to treat the patient within this specific situation of odontogenic deep neck infection.

## Conclusion

Based on the outcomes of the present case and the support of the medical literature, it is possible to infer that accurate diagnosis plays an essential part in the field of maxillofacial surgery and pathology. Furthermore, early medical interventions are valuable to prevent the progression of infection, considering the morphological invasiveness and the aggressiveness of microbiological pathogens. Moreover, dental practitioners must be aware of proper techniques for third molar extraction and follow-up in order to avoid life-threatening situations of deep neck infections.

## Acknowledgments

None.

## Authors’ contributions

AFSJ, GSMR and CFSNA treated the patient in the emergency hospital and developed the initial structure of the manuscript. AF and RFS performed all the drafts up to the final, reviewed the text, and edited for publication. All the authors agreed with the final version of the manuscript.

## Funding

The authors report no funding for this article.

## Competing interests

The authors declare no competing interests with regards to the authorship and/or publication of this article.

## Ethics approval

Not applicable.
